# Delayed union, non‐union and mal‐union in 442 dogs

**DOI:** 10.1111/vsu.13880

**Published:** 2022-08-27

**Authors:** William George Marshall, Barbro Filliquist, Emmanouil Tzimtzimis, Agnieszka Fracka, Jose Miquel, Javier Garcia, Maria Dalla Fontana

**Affiliations:** ^1^ Small Animal Hospital, School of Veterinary Medicine University of Glasgow Glasgow UK; ^2^ Department of Surgical and Radiological Sciences, School of Veterinary Medicine University of California Davis Davis California USA

## Abstract

**Objectives:**

(1) To estimate the prevalence of delayed union, non‐union and mal‐union in canine fractures; (2) to describe fracture, demographic, and treatment characteristics for these outcomes; (3) to identify risk factors for delayed or non‐union.

**Study design:**

Retrospective study.

**Sample population:**

Four hundred and forty two dogs (461 fractures).

**Methods:**

A review was conducted of clinical records and radiographs from 2 teaching hospitals. “Union,” “delayed union,” “non‐union” and “mal‐union” were defined, and fracture, demographic, treatment, and outcome variables described. Differences in proportions or medians between “union,” “delayed union” and “non‐union” were tested using χ^2^ and Mann‐Whitney *U*‐tests for categorical and continuous variables respectively. Potential explanatory variables for “delayed or non‐union” were tested using logistic regression to identify risk factors.

**Results:**

Median radiographic follow up was 53 days (14‐282). Delayed union occurred in 13.9% of fractures (64/461), non‐union in 4.6% (21/461), and mal‐union in 0.7% (3/461). Risk factors for delayed or non‐union were age (OR 1.21, 95% CI 1.12‐1.31); comminuted fracture (OR 4.24, 95% CI 2.4‐7.5); treatment with bone graft (all types) (OR 3.32, 95% CI 1.3‐8.5); surgical site infection (OR 3.24, 95% CI 1.17‐8.97), and major implant failure (OR 12.94, 95% CI 5.06‐33.1).

**Conclusion:**

Older dogs, dogs with comminuted fractures, surgical site infection, or major implant failure were at increased odds of delayed or non‐union. Radius and ulna fractures in toy breed dogs were not at increased odds of delayed or non‐union.

**Clinical significance:**

The identified risk factors should inform fracture planning and prognosticating. The prognosis for radial fractures in toy breeds appears better than historically believed.

## 1 INTRODUCTION

The treatment of canine fractures is generally associated with a favorable outcome as bone is uniquely able to regain its original properties and therefore resume its preoperative function.^1^ Although delayed union, non‐union, and mal‐union are seemingly uncommon, they are associated with morbidity, which may be severe enough to require amputation or euthanasia. In human orthopedics, efforts have focused on determining the incidence or prevalence of these disorders, and predisposing factors.^2^, ^3^, ^4^, ^5^, ^6^, ^7^, ^8^, ^9^, ^10^, ^11^ For example, 8.1% of 3886 patients with fractures of the humerus, femur, or tibia were readmitted within 2 years for management of delayed union, mal‐union or non‐union.^2^ In a large study of 4895 fracture non‐unions occurring within the population of Scotland (5.17 million people), over a 5‐year period, the overall risk of developing non‐union was 1.9%.^3^ The same group identified risk factors for non‐union in a separate study, broadly categorized as mechanical (fracture site instability), host factors (concurrent diseases, medications or lifestyle factors such as smoking), dead bone with a gap at the fracture site, and infection.^4^ Analysis of 309 330 fractures in 18 different bones found that 4.9% developed non‐union, and identified risk factors such as multiple or open fractures, high‐energy injury, and nonsteroidal anti‐inflammatory and opioid medications.^5^

There are few published studies that describe the prevalence of delayed union, non‐union or mal‐union in large populations of dogs. In 1979 Phillips reviewed 284 canine and 298 feline fractures, and described fracture characteristics, etiology, treatment, and outcome.^12^ Of the combined canine and feline fractures, 20 (3.4%) were described as developing non‐union, though the definition of that term was unclear and appeared to include implant failure and refracture. The radius and ulna, and the femur, were most affected (8 cases each), followed by the tibia (3 cases) and humerus (1 case). Non‐union was attributed to technical error, infection, or implant failure in most cases. Delayed union was reported in 1 case, and torsional mal‐union was described in 3 cases (0.5%). In 1984 the authors of a retrospective study of 2825 canine fractures also reported a non‐union prevalence of 3.4% (96 cases), based on clinical and radiographic findings.^13^ Non‐union was found with greatest frequency in the radius and ulna (39 cases) followed by the femur (37 cases), humerus (12 cases), tibia (4 cases), mandible (2 cases), ulna and vertebra (1 case each). Infection was diagnosed (by unspecified means) in 5 non‐unions (5.2%). There was speculation regarding other causes, such as vascular injury and increased movement at the fracture site in younger and more excitable animals. The prevalence of delayed or mal‐union was not given. The authors of a more recent review of 631 canine fractures reported 16.1% delayed union, 8.8% non‐union, and 6.2% mal‐union, although definitions for each disorder were not given.^14^ In summary, the existing literature does not provide reliable contemporary estimates of delayed union, non‐union or mal‐union prevalence in dogs, nor have proposed predisposing factors been rigorously tested.^12^, ^13^, ^14^ Veterinary orthopedic surgeons should know the prevalence of these potentially devastating complications, and understanding which dogs and fractures are most at risk will aid their prevention.

The objectives of this study were: (1) to estimate the prevalence of canine delayed union, non‐union and mal‐union by retrospective review of diverse fracture cases; (2) to describe fracture, demographic, and treatment characteristics in cases of delayed union, non‐union and malunion, and (3) to determine risk factors for delayed or non‐union in canine fractures.

## 2 MATERIALS AND METHODS

### 2.1 Data collection

The clinical record databases of 2 university veterinary teaching hospitals (the University of Glasgow and the University of California, Davis) were searched for all dogs treated for a bone fracture between 2010 and 2019. Inclusion required a known final radiographic outcome. All fractures of the appendicular skeleton were included, as were vertebral body and mandibular fractures. Sacro‐iliac fracture separation, mandibular symphyseal separation, fractures following tibial osteotomies for cruciate disease and fractures treated by salvage surgery (eg, arthrodesis or femoral head and neck excision) were excluded. The case records of each dog and radiographs of each fracture were reviewed. At the University of Glasgow, record and radiograph review was conducted by rotating interns with an interest in surgery (ET, AF, JM, JG and MDF). General progress with the review process, and any specific cases where outcome was uncertain, were discussed with a diplomate orthopedic surgeon (WGM). At the University of California, Davis, the whole review was conducted by a single diplomate orthopedic surgeon (BF). Variables were recorded in a spreadsheet (Microsoft Excel). *Fracture and demographic variables*: Breed, sex, age, bone, whether multiple fractures were sustained simultaneously in distinctly separate bones, fracture configuration, open or closed fracture were recorded. The presence of multiple fractures was recorded even if not all concomitant fractures met the inclusion criteria. For example, a humeral fracture would be included in further analysis, concomitant sacroiliac luxation and pelvic floor fractures would not, but the dog would be considered to have sustained multiple fractures. Similarly, if multiple fractures had been sustained but follow up was not complete for all fractures, the fractures with a final outcome were analyzed and the presence of multiple fractures was recorded. Fractures of “radius and ulna” or “tibia and fibula” were considered single, not multiple fractures. *Treatment variables*: Method of stabilization, use of autogenous or synthetic bone graft and prescription of postoperative antibiotics were recorded. *Outcome variables – complications and bone healing*: Infection was recorded as a complication based on recorded clinical signs, with or without a positive bacterial culture result. Implant failure, any other major or minor complications, timing of each follow‐up assessment postoperatively (in days), and bone healing outcome were recorded. Complications were retrospectively categorized as minor and major as described by Cook et al.,^15^ and in the analysis major complications were subdivided into “implant failure,” “infection,” “implant failure + infection,” and “other.”

### 2.2 Bone healing outcome definitions

For each follow‐up clinical and radiographic assessment an interim or final bone healing outcome was determined by reviewing the clinical records and radiographs. **Interim outcomes**: *Progressing* – a fracture with radiographic evidence of new bone bridging the fracture line and/or callus formation, but further radiographs were recommended. *Delayed* – a fracture that was radiographically uniting more slowly than expected, but it was uncertain whether a delayed or non‐union would result. **Final outcomes**: *Union* – a fracture with radiographic evidence of new bone bridging the fracture line and/or callus formation, within an expected timeframe, and further radiography was not deemed necessary. *Delayed union* – a delayed fracture that, at subsequent follow up, progressed to union. *Non‐union* – a delayed fracture judged unlikely to progress to union without surgical intervention to specifically address failure of bone healing (as opposed to addressing implant failure). Where non‐unions were treated, the outcome of the treatment was also recorded, ie, whether non‐union persisted or union was achieved. *Malunion* – a fracture that healed with abnormal alignment, with or without associated lameness, according to the case record, or as subjectively assessed by the reviewer. Interim outcomes were used to inform the final outcome, but only final outcomes were included in the analysis.

### 2.3 Statistical analysis

Data were assessed for normality visually by the creation of histograms and by using the Shapiro‐Wilks test. Where continuous data were not normally distributed, even following logarithmic transformation, the median and range were calculated, and the Mann‐Whitney *U*‐test was used to examine differences in fracture and demographic variables between bone healing outcome groups. Differences in proportions were tested by comparing delayed and non‐union proportions (separately) with union proportions, using the χ^2^ test. Where there was a statistically significant difference in proportions between delayed *or* non‐union and union, data from delayed *and* non‐union were combined, and compared again with union. Following these analyses, potential risk factors were identified. For the analysis to determine risk factors for delayed healing, data from delayed and non‐union were combined, with the outcome delayed or non‐union = 1 and union = 0. Univariate logistic regression was first performed, entering each potential explanatory variable into the model individually against delayed or non‐union. Explanatory variables that were associated with the outcome at *P* < .2 were included in a multivariate logistic regression model. A stepwise approach was used in the multivariate model to identify those explanatory variables associated with the outcome (*P* < .05). Statistical analyses were performed using Microsoft Excel and MedCalc (MedCalc Software Ltd., Ostend, Belgium).

## 3 RESULTS

Four hundred and sixty‐one fractures in 442 dogs met the inclusion criteria and were included in the analysis (Table 1). The median duration of radiographic follow up across all fractures was 53 days (range 14‐282). In 373 cases (80.9%), union was recorded as the final outcome (this excludes non‐unions that were successfully treated and achieved union). There were 64 cases of delayed union (13.9%), 21 non‐unions (4.6%) and 3 mal‐unions (0.7%). *Fracture variables* for union, delayed union and non‐union are described in Table 1 by bone, with humerus, tibia and fibula, and femur subclassified by fracture type. Of the humeral “non‐condyle” fractures, one with a union outcome affected the proximal humeral physis. All remaining cases affected the humeral diaphysis or distal metaphysis. The femur “distal physis” category included all Salter‐Harris fractures of the distal femur. *Demographic variables* for the final bone healing outcomes union, delayed union and non‐union are described in Table 2. Two dogs had concomitant union and delayed union final outcomes and were excluded from further analysis of demographic variables. *Treatment variables* for the final bone healing outcomes union, delayed union and non‐union are described in Table 3. *Outcome variables*: The number of radiography visits, time to union following fracture stabilization, and complications for union, delayed union and non‐union are given in Table 4. The diagnosis of non‐union was made a median of 92 days following initial treatment (range 46‐228 days). Of the 21 fractures with a non‐union outcome, 9 were successfully revised and progressed to union. In 7 of those cases, recombinant human bone morphogenetic protein‐2 (rhBMP‐2, Pfizer, Cambridge, Massachusetts, USA or Medtronic, Minneapolis, Minnesota, USA) was implanted at the fracture site when the fixation was revised. In 2, a cancellous autograft was used. Of the 12 non‐unions that did not progress to union, revision surgery had been performed in 6 cases, and the last follow‐up radiograph was taken 108 days (median) following the first surgery (range 63‐235 days). Of the 6 cases that were revised, 3 were treated with rhBMP‐2 and in the remaining 3 no bone graft or substitute was used. There were no differences in the proportion of minor or major complications occurring in non‐unions that were successfully revised versus those that were not. Where proportions were different from union for delayed union or non‐union but not both, the combined difference was tested (Table 5). We did not perform this step for the different bone graft materials, as when all materials were combined, differences in proportions were found for both delayed and non‐union versus union. There were 3 mal‐unions – 1 humeral shaft fracture, 1 lateral humeral condylar fracture, and 1 fracture of the tibia and fibula. All were treated with open reduction and internal fixation, all recorded implant failure as a postoperative complication (and cause of the mal‐union), and in no case was revision surgery performed. Because only 3 cases were identified, there was no further analysis of mal‐union as a final outcome. The χ^2^ and Mann‐Whitney *U*‐tests identified the following potential explanatory variables for bone healing outcome, which were then analyzed using logistic regression: *Fracture variables*: humerus Y or T, humerus non‐condyle, ilium, comminuted, open. *Dog variables*: age, male castrated. *Treatment variables*: postoperative antibiotics, all bone grafts, open reduction and internal fixation. *Outcome variables*: infection, implant failure, infection + implant failure (all major complications). Following univariate logistic regression, ilium and infection + implant failure were excluded from the multivariate analysis. The remaining variables were entered into the multivariate logistic regression model. Comminuted, age, bone graft, infection and implant failure were associated with the outcome (Table 6). The *P* value for overall model fit was <.0001 and the Hosmer and Lemeshow test showed acceptable logistic regression model fit (*χ* ^2^ = 10.79; *P* = .21).

**TABLE 1:** Characteristics of fractures with union, delayed union, and non‐union

Final bone healing outcome	Union	Delayed union	Non‐union
Total number fractures (%)	373 (80.9)	64 (13.9)	21 (4.6)
Fractures by bone/fracture type (number and %)			
Mandible	4 (1.1)	0	1 (4.8)
Vertebrae	2 (0.5)	0	0
Metacarpal	4 (1.1)*	0	2 (9.5)*
Radius	4 (1.1)	0	0
Ulna	3 (0.8)	2 (3.1)	1 (4.8)
Radius and ulna	92 (24.7)	21 (32.3)	4 (19.0)
Humerus condyle – lateral	37 (9.9)	4 (6.2)	1 (4.8)
Humerus condyle – medial	5 (1.3)	0	0
Humerus condyle – Y or T	8 (2.1)*	6 (9.4)*	1 (4.8)
Humerus non‐condyle	15 (4.0)*	9 (14.1)*	3 (14.3)*
Metatarsal	8 (2.1)	0	1 (4.8)
Tarsal bones I, II, or III	1 (0.3)	0	0
Calcaneus	3 (0.8)	0	0
Talus	2 (0.5)	0	0
Fibular malleolus	2 (0.5)	0	0
Tibial and fibular malleoli	3 (0.8)	1 (1.5)	0
Tibia – tuberosity	18 (4.8)	1 (1.5)	0
Tibia	20 (5.4)	1 (1.5)	0
Tibia and fibula	34 (9.1)	6 (9.2)	3 (14.3)
Fibula	1 (0.3)	0	0
Patella	1 (0.3)	0	0
Femur – diaphysis/metaphysis	42 (11.3)	12 (18.8)	4 (19.0)
Femur – distal physis	18 (4.8)	1 (1.6)	0
Femur – proximal physis	6 (1.6)	0	0
Femur – neck	6 (1.6)	0	0
Acetabulum	2 (0.5)	0	0
Ilium	32 (8.6)*	0*	0
Open fracture (number and %)	17 (4.6)*	13 (20.0)*	5 (23.8)*
Comminuted fracture (number and %)	68 (18.2)*	33 (51.6)*	13 (61.9)*

* Statistically significant differences in proportions (*P* < .05, delayed or non‐union versus union).

**TABLE 2:** Demographics of dogs with fracture union, delayed union, and non‐union

Final bone healing outcome	Union	Delayed union	Non‐union
Number of dogs	358	61	20
Breed (number and %)			
Australian cattle dog	5 (1.4)	1 (1.6)	0
Australian shepherd	11 (3.1)	1 (1.6)	0
Border collie	10 (2.8)	2 (3.3)	1 (5.0)
Boston terrier	5 (1.4)	0	0
Boxer	8 (2.2)	0	0
Chihuahua	16 (4.5)	2 (3.3)	1 (5.0)
Cocker Spaniel	9 (2.5)	3 (4.9)	1 (5.0)
Crossbreed	77 (21.5)	15 (24.6)	3 (15.0)
French Bulldog	7 (2.0)	0	0
German Shepherd	11 (3.1)	3 (4.9)	0
Husky	5 (1.4)	1 (1.6)	0
Italian Greyhound	8 (2.2)	2 (3.3)	0
Jack Russell Terrier	6 (1.7)	1 (1.6)	0
Labrador Retriever	34 (9.5)	2 (3.3)	2 (10.0)
Lurcher	5 (1.4)	1 (1.6)	0
Miniature Pinscher	5 (1.4)	0	0
Pit Bull Terrier	10 (2.8)	1 (1.6)	0
Pomeranian	9 (2.5)	1 (1.6)	1 (5.0)
Shih‐Tzu	7 (2.0)	0	0
Springer Spaniel	13 (3.6)	2 (3.3)	1 (5.0)
Toy Poodle	9 (2.5)	2 (3.3)	1 (5.0)
Yorkshire Terrier	8 (2.2)*	3 (4.9)	2 (10.0)*
Other terrier breeds	15 (4.2)	2 (3.3)	1 (5.0)
Other breed	65 (18.2)	16 (26.2)	6 (30.0)
Sex (number and %)			
Female	98 (27.2)	12 (19.0)	3 (15.0)
Female spayed	86 (23.9)	16 (25.4)	7 (35.0)
Male	78 (21.7)	8 (12.7)	7 (35.0)
Male castrated	98 (27.2)*	27 (42.9)*	4 (20.0)
Number of dogs with >1 bone fractured simultaneously (%)	54 (15)	6 (9.5)	6 (30.0)
Median dog age in years (range)	1 (0.1–14)*	4 (0.1–15.1)*	8 (0.2–13)*

* Statistically significant differences in proportions or median (*P* < .05, delayed or non‐union versus union).

**TABLE 3:** Treatment variables in dogs with fracture union, delayed union, and non‐union

Final bone healing outcome	Union	Delayed union	Non‐union
Total number fractures	373	64	21
Fracture treatment method (%)			
ORIF	302 (81.0)*	44 (68.8)*	14 (66.7)
MIPO	13 (3.5)	5 (7.8)	1 (4.8)
MIO	14 (3.8)*	3 (4.7)	4 (19.0)*
ESF	36 (9.7)	8 (12.5)	2 (9.5)
ESF + ORIF	7 (1.9)	2 (3.1)	0
External coaptation	1 (0.3)	1 (1.6)	0
Cage rest	0	1 (1.6)	0
Bone graft or substitute used in first surgery (%)			
Cancellous	9 (2.4)*	6 (9.4)*	2 (9.5)
Cancellous + allograft	0	1 (1.6)	0
Allograft	3 (0.8)*	5 (7.8)*	1 (4.8)
BMP‐2	1 (0.3)	0	0
All bone grafts or substitutes	13 (3.5)*	12 (18.8)*	3 (14.3)*
Postoperative antimicrobials (%)	165 (44.2)*	42 (65.6)*	11 (52.4)

Abbreviations: ESF, external skeletal fixation; MIO, minimally invasive osteosynthesis; MIPO, minimally invasive plate osteosynthesis; ORIF, open reduction and internal fixation.

* Statistically significant differences in proportions (delayed or non‐union versus union).

**TABLE 4:** Outcome variables: median number of radiography visits, time to union and complication proportions in dogs with fracture union, delayed union, and non‐union

Final bone healing outcome (number of fractures)	Union (373)	Delayed union (64)	Non‐union (21)
Median number of follow‐up radiography visits (range)	1 (1–3)*	2 (2–6)*	2 (2–5)*
Median time to union in days (range). (9/21 non‐union cases achieved union)	48 (14–147)*	106.5 (27–273)*	208 (104–282)*
Minor complications (%)	51 (13.7)	7 (10.9)	2 (9.5)
Major complications − implant failure (%)	10 (2.7)*	15 (23.4)*	7 (33.3)*
Major complications − infection (%)	17 (4.6)*	8 (12.5)*	4 (19.0)*
Major complications − implant failure + infection (%)	0*	5 (7.8)*	1 (8.3)*
Major complications − other (%)	14 (3.8)	0	1 (8.3)

* Statistically significant differences in proportions or median (*P* < .05, delayed or non‐union versus union).

**TABLE 5:** Fracture, demographic, and treatment variables where proportions differed between union versus delayed union *or* non‐union. Union versus delayed union *and* non‐union combined was tested to eliminate spurious results

Final bone healing outcome (number of fractures/dogs)	Union (373/358)	Delayed union and non‐union (85/81)
Ilium	32 (8.6)*	0*
Humerus condyle – Y or T	8 (2.1)*	7 (8.2)*
Metacarpal	4 (1.1)	2 (2.4)
Yorkshire terrier	8 (2.2)	5 (6.2)
Male castrated	98 (27.2)*	31 (38.3)*
Postoperative antimicrobials (%)	165 (44.2)*	53 (62.4)*
MIO	14 (3.8)	7 (8.2)
ORIF	302 (81.0)*	58 (68.2)*

Abbreviations: MIO, minimally invasive osteosynthesis; ORIF, open reduction and internal fixation.

* Statistically significant differences in proportions (*P* < .05, delayed or non‐union versus union).

**TABLE 6:** Factors associated with delayed fracture healing or non‐union in dogs

	OR	95% CI	*P*
Age	1.21	1.12–1.31	<.0001
Comminution	4.24	2.40–7.50	<.0001
All bone grafts	3.32	1.30–8.50	.01
Infection	3.24	1.17–8.97	.02
Implant failure	12.94	5.06–33.10	<.0001

## 4 DISCUSSION

Defining fracture union is a challenge. Union is a gradual and lengthy process rather than an endpoint, and some threshold must be imposed to define and study it.^16^ Here we used a broad and subjective threshold for the definition of union – satisfactory radiographic progression of bone formation at the fracture site within an expected timeframe, and further radiographs were not taken. The unblinded and subjective assessment of clinical and radiographic findings is a common way of assessing union in human traumatology studies, although it clearly may be subject to bias.^17^ Defining and distinguishing between delayed and non‐union is also fraught with difficulty. In both, the fracture has not united within the expected timeframe, which seems inherently subjective and variable between clinicians, patients (or dogs), bones, fracture configurations, and etiologies. In both delayed and non‐union, implant failure may occur, requiring revision surgery.^18^ In defining delayed and non‐union here, we made a distinction between revision surgery to treat implant failure alone in delayed union, versus revision surgery to address a perceived cessation of bone healing in non‐union, but this was not always straightforward, and others may have defined some of our cases differently.

The median postoperative time to diagnosis of non‐union was around 3 months, but the lower end of the range was 46 days, which seems more in line with delayed union. In that case, further radiographs were taken 84 days post‐op, confirming a perhaps premature prediction of non‐union. Strictly distinguishing between delayed and non‐union may be illogical,^19^ and there is considerable overlap between risk factors for delayed and non‐union.^6^ For these reasons, the outcomes delayed and non‐union were combined in the analyses performed here. We did not describe the traditional radiographic classification of the non‐unions in this report (hypertrophic, oligotrophic, atrophic). The value of this system has been questioned, as it was developed when many fractures were managed nonsurgically, it has not been validated, and the assumption that atrophic non‐unions are avascular has been disproven.^7^, ^20^, ^21^ The accepted general definition of mal‐union as a fracture that has healed in a nonanatomical position is clearer, although in human orthopedics detailed criteria have been developed for individual bones.^22^

Using the definition given, the prevalence of canine fracture non‐union found here (4.6%) lies between the 3.4% and 8.1% reported previously.^12^, ^13^, ^14^ The prevalence of delayed union (13.2%) is comparable with Galladah et al.^14^ (16.1%), the prevalence of mal‐union is lower (0.7% vs. 6.2%). Compared with non‐union prevalence, delayed and mal‐union prevalence is less commonly found in the human orthopedic literature. A 32.4% delayed union prevalence was reported for human proximal humeral fractures (and 8.2% non‐union).^8^ Figures for the prevalence of mal‐union in the human literature range widely depending on the specific bone and the defining criteria used.^22^ The median time to union in our study was 48 days, which is in line with previous reports of 7.5 weeks and 64 days (mean) for union of fractures treated by open reduction and internal fixation.^23^, ^24^ The non‐unions that did eventually heal after revision surgery took a total median time of 208 days from injury to achieve union. A previous case series of non‐unions reported a similar mean healing time (from the time of original injury) of 257.8 days.^25^

In the multivariate analysis no individual bone was associated with bone healing outcome, but we found the proportion of humeral diaphyseal and Y‐fractures that progressed to delayed or non‐union was greater than the proportion that healed uneventfully. This disagrees with previous reports where the radius and ulna showed the greatest frequency of delayed healing.^12^, ^13^ Neither study compared the proportion of fractures that showed delayed or non‐union with union, and frequency therefore cannot be interpreted as prevalence. In our study, there was no association between delayed or non‐union and any breed. The significantly (*P* < 0.05) greater proportion of Yorkshire terriers with non‐union appears to be spurious as this was not found for delayed union, or when delayed and non‐union were combined. It is often stated that toy breed dogs with fractures of the radius and ulna are at increased risk of delayed and non‐union,^26^, ^27^ but the evidence for this appears weak, and our results do not support it. Recent advances in fracture fixation techniques may have improved outcomes for radius and ulna fractures in toy breed dogs. Although not significant in the logistic regression analysis, delayed or non‐union was not recorded for any ilial fractures, and considering experimental evidence for the importance of surrounding muscle in bone healing,^28^ we speculate that a generous muscle envelope may make ilial fractures more likely to heal uneventfully.

The proportions of open fractures that developed delayed or non‐union were significantly (*P* < 0.05) increased, though “open” was not confirmed as a risk factor in the multivariate analysis. Open fractures are at greater risk of non‐union in human orthopedics, and patients with multiple injuries or fractures are also considered at greater risk of non‐union.^5^, ^9^ In our study, multiple fractures were not associated with delayed or non‐union. The low frequency of delayed or non‐union where multiple bones were fractured (6 cases each) may have caused a Type II error, and reflects the reality of veterinary clinical decision making—animals with severe multiple fractures are often euthanized on the grounds of treatment cost or prognosis.

Comminution, age, the use of bone graft, infection, and implant failure all increased the odds of the outcome delayed or non‐union. Comminuted fractures were 4 times more likely to show delayed or non‐union. Comminuted fracture patterns and high‐energy injuries are risk factors for non‐union in human traumatology.^5^, ^10^, ^11^ We can speculate that the high energy trauma typically associated with comminuted fractures may cause a greater degree of injury to the soft tissue envelope, compromising vascularity, or because comminuted fractures are more reliant on orthopedic implants for their stability they may be more at risk of implant failure. The authors could find scant evidence regarding the precise mechanism of association between comminution and delayed healing.

We found that the odds of delayed or non‐union in dogs increased with increasing age. The negative effect of increasing age on bone healing has been studied in laboratory rats and mice.^29^ A number of mechanisms contribute to slower bone healing in older animals: impaired callus bridging and increased bone resorption through increased osteoclast activity, decreased osteoblast response to osteogenic stimuli, and delayed chondroblast differentiation and maturation during endochondral ossification.^29^ In a study of fracture non‐union in domestic cats, the median age of those developing non‐union was 5 years, versus 2 years for control (union) cats.^30^ In humans the situation appears different; in one study fractures in patients between the ages of 35 and 44 had the greatest risk of developing non‐union. Low‐energy fractures of metaphyseal bone (eg, distal radial fractures), which occur commonly in older patients, may have better healing potential than high‐energy injuries seen in younger patients (eg open tibial fractures following a motorcycle accident).^7^ Bone grafting was associated with increased odds of delayed or non‐union, which is counterintuitive. It is unlikely that bone grafting contributed to the delay in bone healing but rather the decision to use bone graft was based on the perceived risk of delayed healing.

We found no difference in the prevalence of minor complications between union, delayed and non‐union, but major implant failure occurred in a third of non‐union cases and nearly a quarter of delayed unions, versus 2.7% of unions. Implant failure was associated with a more than 12 times increased odds of delayed or non‐union, although it is possible that some cases of delayed or non‐union had another cause (eg, infection), and implant failure was rather an effect. Fracture non‐union in humans is associated with inadequate fixation or stabilization,^4^ and implant failure will create a suboptimal strain environment at the fracture site, resulting in delay or failure of bone healing.^31^ Surgical site infection was diagnosed in 12.5% and 19.0% of delayed and non‐unions respectively, versus 4.6% of unions, and infection was associated with threefold increased odds of delayed bone healing. Human studies have also identified infection as a significant factor in the development of fracture non‐union.^4^, ^6^, ^11^ Infection of bone retards fracture healing through inflammation, apoptosis of osteoblasts and increased osteoclast activity,^32^ though in our retrospective study not all surgical site infections were confirmed as osteomyelitis.

This study is limited by its retrospective nature, and by the difficulty inherent in defining bone healing disorders. Clinical records and radiographs were reviewed at 2 different institutions, by 7 different clinicians, all of whom may have categorized outcomes slightly differently. The goal of the study was to determine risk factors for delayed bone healing across a diverse population of fractures, but the relative importance of the factors identified may vary between different bones. Other possible risk factors for delayed or non‐union could be explored in future studies, such as duration of nonsteroidal anti‐inflammatory drug administration postoperatively, and the quality of surgical repair in terms of apposition, alignment, and apparatus. We did not attempt to examine the relationship between the appropriate execution of fracture stabilization, and implant failure, or delayed union, mal‐union or non‐union, and recognize this as a limitation. Defining criteria for assessing quality of repair would be challenging, and is perhaps something for a future study.

In conclusion, we have estimated the prevalence of non‐union (4.6%), delayed union (13.9%), and malunion (0.7%) in a heterogeneous population of 442 dogs with 461 bone fractures. We have found increased odds of delayed or non‐union in older dogs, comminuted fractures, fractures treated with bone graft materials, and in those that developed postoperative surgical site infection or implant failure. Our study does not support the traditionally held belief that fractures of the radius and ulna in toy breed dogs are more likely to develop delayed or non‐union.

## CONFLICT OF INTEREST

The authors declare no conflicts of interest related to this report.
